# PRMT5 Selective Inhibitor Enhances Therapeutic Efficacy of Cisplatin in Lung Cancer Cells

**DOI:** 10.3390/ijms22116131

**Published:** 2021-06-07

**Authors:** Khuloud Bajbouj, Rakhee K. Ramakrishnan, Maha Saber-Ayad, Hany A. Omar, Narjes Saheb Sharif-Askari, Jasmin Shafarin, Adel B. Elmoselhi, Ahmed Ihmaid, Suhib AlHaj Ali, Abdulla Alalool, Reem Abdullah, Qutayba Hamid

**Affiliations:** 1 College of Medicine, University of Sharjah, Sharjah 27272, United Arab Emirates; kbajbouj@sharjah.ac.ae (K.B.); u16101422@sharjah.ac.ae (R.K.R.); nsharifaskari@sharjah.ac.ae (N.S.S.-A.); amoselhi@sharjah.ac.ae (A.B.E.); ahmed.m.h.humaid@gmail.com (A.I.); suhaib_al-hajali@hotmail.com (S.A.A.); abdullaalalool@gmail.com (A.A.); reem.mabdullah@gmail.com (R.A.); 2 Sharjah Institute for Medical Research, University of Sharjah, Sharjah 27272, United Arab Emirates; hanyomar@sharjah.ac.ae (H.A.O.); jsalam@sharjah.ac.ae (J.S.); 3 College of Medicine, Cairo University, Cairo 11956, Egypt; 4 College of Pharmacy, University of Sharjah, Sharjah 27272, United Arab Emirates; 5 Department of Physiology, Michigan State University, East Lansing, MI 48824, USA; 6 Meakins-Christie Laboratories, Research Institute of the McGill University Health Center, Montreal, QC H4A 3J1, Canada

**Keywords:** PRMT5, lung cancer, epigenetics, cisplatin, histone, HBEpC, A549 and DMS 53

## Abstract

As a therapeutic approach, epigenetic modifiers have the potential to enhance the efficacy of chemotherapeutic agents. Protein arginine methyltransferase 5 (PRMT5), highly expressed in lung adenocarcinoma, was identified to be involved in tumorigenesis. In the current study, we examined the potential antineoplastic activity of PRMT5 inhibitor, arginine methyltransferase inhibitor 1 (AMI-1), and cisplatin on lung adenocarcinoma. Bioinformatic analyses identified apoptosis, DNA damage, and cell cycle progression as the main PRMT5-associated functional pathways, and survival analysis linked the increased PRMT5 gene expression to worse overall survival in lung adenocarcinoma. Combined AMI-1 and cisplatin treatment significantly reduced cell viability and induced apoptosis. Cell cycle arrest in A549 and DMS 53 cells was evident after AMI-1, and was reinforced after combination treatment. Western blot analysis showed a reduction in demethylation histone 4, a PRMT5- downstream target, after treatment with AMI-1 alone or in combination with cisplatin. While the combination approach tackled lung cancer cell survival, it exhibited cytoprotective abilities on HBEpC (normal epithelial cells). The survival of normal bronchial epithelial cells was not affected by using AMI-1. This study highlights evidence of novel selective antitumor activity of AMI-1 in combination with cisplatin in lung adenocarcinoma cells.

## 1. Introduction

Lung cancer is one of the most prevalent cancers worldwide. According to the 2018 report of the WHO International Agency for Research on cancer, lung cancer is the most commonly diagnosed cancer, with 2.09 million cases (11.6% of the total cases) in both sexes, and the leading cause of cancer death (18.4% of the total cancer deaths), [1]. Despite a declining trend in the incidence of lung cancer in the last two decades, it remains the leading cause of cancer mortality among both men and women, with a vicious 5-year relative survival of only 18% [2]. At present, platinum-based therapy, including cisplatin and carboplatin are the most common regimens used for the treatment of lung cancer. Despite multimodal current therapy, instances of recurrence and metastasis are high in lung cancer, urging the development of novel therapeutic options.

The carcinogenesis of lung cancer is steered by the acquisition of genetic and epigenetic alterations. Epigenetic modifications, such as DNA methylation, histone modifications, and non-coding RNA expression, have been extensively implicated in lung cancer [3]. The frequency of such epigenetic events increases with the progression of the disease. The hypermethylation of the promoter region of tumor suppressor genes is a hallmark of lung cancer [4,5]. The methylation of promoters of specific genes (e.g., TAC1, HOXA7, and SOX17) in sputum and blood are strongly correlated with the risk of lung cancer. Therefore, changes in the pattern of DNA methylation is of special interest in human lung cancer as it identifies potential markers for early molecular detection. Furthermore, the reversible nature of epigenetic aberrations has made its correction an attractive therapeutic approach. 

A family of nine protein arginine methyltransferases (PRMTs) is responsible for the catalysis of arginine methylation; a post-translational modification implicated in the regulation of numerous cellular processes. This includes gene transcription, signal transduction, mRNA splicing, and DNA damage response. PRMT5 is the primary enzyme responsible for the symmetric dimethylation of arginine residues on target proteins [6]. It plays a functional role in diverse processes, including transcriptional regulation and cell cycle regulation [4,6,7]. In mammalian cells, PRMT5 shuttles between the cytoplasm and nucleus and is responsible for the methylation of both histone as well as non-histone proteins. The PRMT5-mediated arginine dimethylation of histones, H3 and H4, alters the chromatin structure to induce transcriptional repression [8,9].

In the context of cancer, PRMT5 is generally regarded as a tumor initiator and promoter of tumor growth. The subcellular localization of PRMT5 is dynamically regulated during cellular differentiation and regulates PRMT5 function. PRMT5 contributes to prostate tumorigenesis by localizing predominantly in the cytoplasm to promote cell growth in a PRMT5 methyltransferase activity-dependent manner [10]. PRMT5 acts as a transcriptional repressor by suppressing the expression of tumor suppressor genes and the dysregulation of PRMT5 has been reported in multiple cancer types. For instance, PRMT5 mediates the methylation of p53, thereby, impeding its ability to restrict cell growth and promote apoptosis [11]. Increased PRMT5 activity triggers cyclin D1/CDK4 kinase-dependent neoplastic growth [12]. Elevated expression levels of PRMT5 and its enhanced methyltransferase activity have also been implicated in the proliferation of lung cancer cells [13]. PRMT5 has also been shown to accelerate lung cancer progression and metastasis via the histone methylation of the miR-99 family and in turn, activating the Erk1/2 and Akt pathway through FGFR3 signaling [14]. In addition, PRMT5 expression correlated with the epithelial–mesenchymal transition in lung cancer cell lines and the subcellular localization of PRMT5 varied depending on the histologic grade of lung adenocarcinoma suggesting PRMT5 as a marker of prognostic value [15]. Therefore, PRMT5 inhibition is a promising therapeutic target with the potential to sensitize tumors to chemotherapy [16].

Arginine methyltransferase inhibitor 1 (AMI-1) is a small molecule inhibitor of PRMTs, which demonstrated potent anti-tumor activity against hepatocellular carcinoma by inhibiting type II PRMT5 activity [17]. In this study, we aim to explore the effect of the combination of PRMT5 inhibitor (AMI-1) and cisplatin on cell viability in an in vitro lung cancer model, compared with normal bronchial epithelial cells. To determine the association between PRMT5 gene expression level and survival of lung cancer patients, and to identify the major functional pathways related to PRMT5 activity in lung adenocarcinoma, we carried out bioinformatic analyses using the publicly available data. We also investigated the mechanism of this effect focusing on the induction of apoptosis, histone methylation, and cell cycle arrest. 

## 2. Results

### 2.1. Bioinformatics

**Figure 1:**
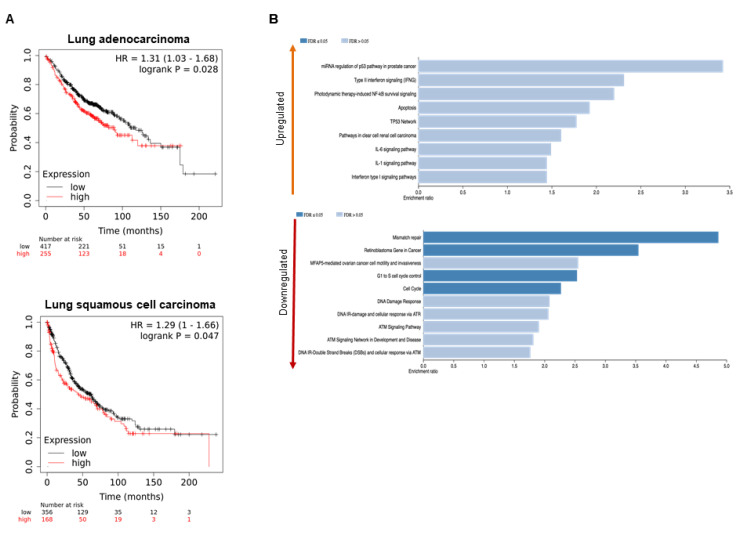
Bioinformatics analyses to determine the prognostic value of PRMT5 and its major functional pathways in lung cancer (**A**) Kaplan–Meier survival analysis of overall survival revealed that high PRMT5 expression was significantly associated with worse overall survival (OS) in both lung cancer subtypes of adenocarcinoma and squamous cell carcinoma. (**B**) Using a publicly available dataset (GSE56757), the differentially expressed genes between PRMT5-silenced and control A549 lung cancer cells were identified and entered into WebGestalt tool to determine the top upregulated and downregulated functional pathways.

The survival analyses of publicly available lung cancer data revealed that an increased level of *PRMT5* gene expression was significantly linked to worse overall survival (OS) in lung adenocarcinoma with the pooled hazard ratio (HR) of 1.31 (95% confidence interval [CI] 1.03–1.68, *p*-value of 0.028). This marker was also associated with worse OS in the second lung cancer subtype of squamous cell carcinoma with the pooled HR of 1.29 (95% CI 1–1.66, *p*-value of 0.047). Figure 1A shows the survival analysis plot for both lung cancer subtypes. Bioinformatics analyses were performed to determine the major pathways associated with PRMT5 and to highlight the overall role of PRMT5 in the pathogenesis of lung adenocarcinoma. The differentially expressed genes between PRMT5-silenced and control A549 lung cancer cells were entered into the WebGestalt tool to determine the major PRMT5-associated pathways under the functional database of Wikipathway cancer. Figure 1B shows the top pathways linked to both the upregulated and downregulated genes. Among the identified pathways linked to PRMT5 knockdown, increase in apoptosis and decrease in DNA damage, retinoblastoma gene, and cell cycle (G1 to S cell cycle control) pathways were selected for further validation in this study.

### 2.2. Cell Viability Was Reduced by Combined Treatment with Cisplatin and AMI-1

**Figure 2:**
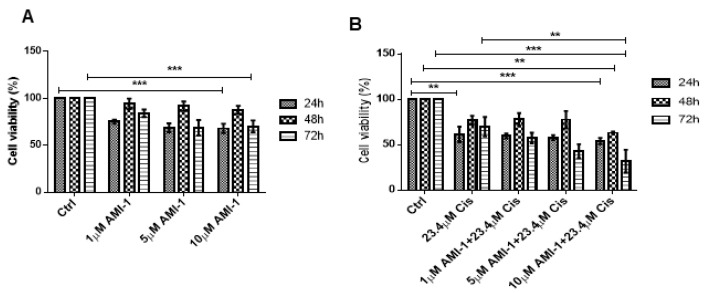
AMI-1 alone and in combination with cisplatin reduces the viability of A549 cells. The cellular viability of A549 cells upon treatment with AMI-1.; cisplatin or both was measured using MTT colorimetric assay. (**A**) A549 cells were exposed to several AMI-1 concentrations (1, 5, and 10 µM) and viability was analyzed at 24, 48 and 72 h. AMI-1 inhibited cellular viability at concentrations of 10 µM when treated for 48 h. (**B**) Cells were treated with cisplatin alone and in combination with 1, 5 and 10 µM AMI-1., and viability was assessed at 24, 48 and 72 h. 10 μM AMI-1 in combination with IC_50_ of cisplatin (23.4 µM) significantly inhibited cell survival at 72 h. Data are normalized to untreated control (Ctrl) and is representative of two independent experiments. * represents a statistically significant change in viability between the indicated treatment groups at given time points. ** *p* < 0.01.; *** *p* < 0.001.

To explore the role of PRMT5 in A549 cell proliferation, we tested different concentrations of PRMT5 inhibitor AMI-1 (1, 5 and 10 μM) as a single treatment and in combination with cisplatin at its IC_50_ concentration. We tested the viability using the MTT assay at 24, 48, and 72 h. The dose response curve of AMI is shown in Figure 2A. Cell viability was significantly reduced by a combined effect of AMI-1 (at 10 μM) and cisplatin, Figure 2B. 

To detect the type of interaction between AMI-1 and cisplatin on cell viability, the interaction observed in all of the tested concentrations of each combination was calculated. Using Calcusyn software, the CI calculated for combined Cisplatin (IC50) and AMI-1 (5 and 10 μM) at 72 h was 0.9 and 0.6, respectively, denoting a synergistic effect of AMI-1 at the dose of 5 and 10 μM (Figure 2B, [18]).

### 2.3. Combination Treatment Demolished Histone 4 Methylation

**Figure 3:**
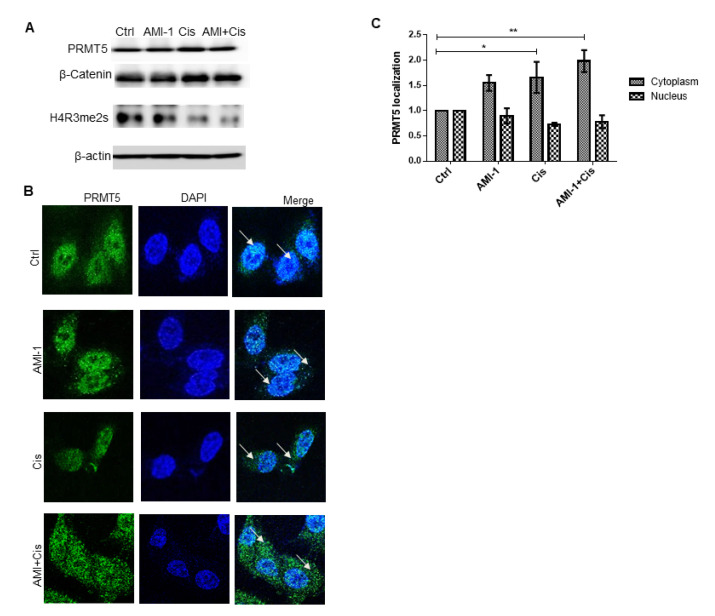
Protein profiling of PRMT5 and its downstream targets; β-catenin and H4R3me2s.; in A549 cells. A549 cells were treated with AMI-1.; cisplatin or both and harvested after 48 h of treatment. (**A**) Representative Western blot of PRMT5; β-catenin.; and H4R3me2s levels in A549 cells after administration of AMI-1 and/or cisplatin for 48 h. ß-actin was used as a loading control. AMI-1 (10 μM) in combination with cisplatin markedly reduced the methylation of histone H3. (**B**) Immunofluorescence staining of A549 cells exposed to different treatment conditions left untreated than were stained for DNA (DAPI; blue).; PRMT5 (green). Arrows indicate subcellular localization of PRMT5 in A549 lung cancer cells under 40× objective lens. (**C**) Bar graph showing the PRMT5 cellular localization in cells treated with AMI-1 and cisplatin alone or in combination in comparison to untreated cells. (*) Represents the statistically significant change in viability between the indicated treatment groups at given time points. ** *p* < 0.01.

Western blot analysis was performed on the whole-cell lysates derived from A549 cells treated with AMI-1, cisplatin or a combination of both using antibodies against PRMT5, β-catenin, and H4R3me2s. The combined effect of PRMT5 inhibitor (AMI-1) with cisplatin did not show a decrease in PRMT5 and β-catenin levels. However, combination treatment resulted in the remarkable methylation reduction in PRMT5-downstream target H4R3me2s after 48 h of treatment (Figure 3A). The sub-cellular localization of PRMT5 plays a major role in tumor progression; therefore, we investigated its location in untreated cells and in ones exposed to different treatments. Figure 3B showed that PRMT5 was predominantly found in the nucleus. However, combination treatments led to translocation into the cytoplasm. This shuttling supports the observed H4R3me2s demethylation (Figure 3B,C).

### 2.4. G1 Cell Cycle Arrest Was Induced after AMI-1 and Cisplatin Administration

**Figure 4:**
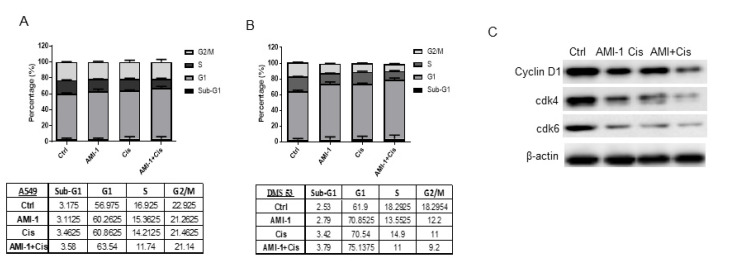
AMI-1 in combination with cisplatin induces G1 cell cycle arrest in lung cancer cells. The cell cycle progression in A549 and DMS 53 cells upon treatment with AMI-1; cisplatin or both for 48 h was measured by propidium iodide (PI) staining and analyzed using flow cytometry. (**A**,**B**) Bar graph showing the percentage of cells in sub-G1; G1; S; and G2/M representative of flow cytometric analysis of A549 cells (**A**) and DMS 53 cells (**B**) treated with AMI-1 and cisplatin alone or in combination with cisplatin indicating the different phases of the cell cycle. Percentage of cells occupying sub-G1; G1; S; and G2/M phases of the cell cycle were determined using the cell cycle platform of FlowJo software. Data here are representative of two independent experiments. Tables represent values of individual cell populations in each cell cycle phase. (**C**) Representative Western blot of cyclin D1; cdk4; and cdk6 levels in A549 cells after administration of AMI-1 and/or cisplatin. ß-actin was used as the loading control.

Combination treatment with 10 µM AMI-1 and IC_50_ of cisplatin significantly reduced viable cell percentages at 48 h. Treatment with both drugs at 48 h led to a cell growth arrest. As shown in Figure 4A, 48 h of both drug administration hindered the ability of A549 cells to recycle as the G1 occupying cell percentage was increased after combination treatment. Combination treatment induced similar G1 cell cycle phase arrest in lung cancer cell line DMS 53 (Figure 4B). The observed G1 arrest was evident by the reduced protein levels of G1 cell cycle phase regulators; cyclin D1, cdk4, and cdk6 (Figure 4C), indicating defective transition and progression abilities of the cells after combination treatment. There was a low percentage of cell death induction after 48 h of treatment with both drugs that was evident by the number of cells occupying SubG1 (Figure 4A,B). The G1 arrest was confirmed by the reduced protein levels of G1 cell cycle phase regulators; cyclin D1, cdk4, and cdk6 (Figure 4C), indicating defective transition and progression abilities of the cells after combination treatment. There was a low percentage of cell death induction after 48 h of treatment with both drugs that was evident by the number of cells occupying SubG1 (Figure 4A,B).

### 2.5. Selective Apoptosis Was Promoted in Lung Adenocarcinoma Cells and Not Human Bronchial Epithelial Cells

**Figure 5:**
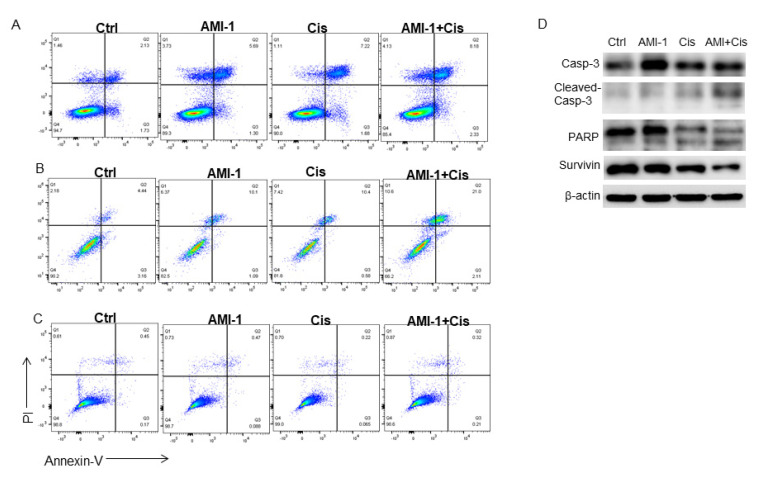
AMI-1 in combination with cisplatin augments apoptosis in lung cancer cells but not in HBEpC cells. A549; DMS 53 and HBEpC cells were cultured in the presence of AMI-1; cisplatin or both for 72 h. (**A**) A549 cells were stained with annexin V-FITC/PI and apoptosis read using flow cytometry. Flow cytometric acquired data were analyzed using the FlowJo software. (**B**) DMS 53 cells were stained with annexin V-FITC/PI and apoptosis read using flow cytometry. (**C**) HBEpC cells were stained with annexin V-FITC/PI and apoptosis read using flow cytometry Flow cytometric plots demonstrate 10 μM AMI-1 in combination with cisplatin selectively induced apoptosis post 72 h of treatment in both lung cancer cells, not HBEpC cells. Data are from one representative experiment out of at least three. (**D**) Representative Western blot of caspase-3; PARP; and survivin levels in A549 cells after administration of AMI-1 and/or cisplatin for. ß-actin was used as the loading control.

To investigate the effect of AMI-1 on lung cancer cell viability compared to normal epithelial cells, both lung cancer cells and HBEpC cells were treated with AMI-1, Cis, or a combination of two drugs, and apoptosis was determined at 72 h by annexin V/propidium iodide staining, as shown in Figure 5A–C. To investigate cellular mechanisms associated with the observed cell death, we examined caspase-3 activity during different treatment conditions. Caspase-3 cleavage was mostly found after combination treatment and the activity was confirmed by the cleavage increase in its downstream target, PARP (Figure 5D). On the other hand, combination treatment did not show any remarkable effect on normal human bronchial epithelial cells (Figure 5C).

## 3. Discussion

The current study shows a novel selective antitumor activity of the epigenetic modifier, PRMT5 inhibitor AMI-1, additive to cisplatin in lung adenocarcinoma cells. The micromolar concentration of AMI-1 mediates anti-survival effect through G1 arrest and reduction in histone-4 dimethylation. Interestingly, AMI-1 is cytoprotective on normal epithelial cells.

Epigenetic targeting drugs can potentiate the effect of conventional chemotherapeutic agents in solid tumors [19]. The overexpression of PRMT5 has been observed in solid and hematological malignancies, e.g., mantle cell lymphoma (MCL) which depends on PRMT5 activity for their survival [20]. PRMT5 mediates the methylation of several proteins, including E2F-1. Among the E2F family of transcription factors, E2F-1 has been implicated in opposing fates of cell cycle progression and apoptosis, depending on the cellular context [21]. The dysregulation of E2F activity occurs in many tumors as a result of the inactivation of the retinoblastoma tumor suppressor pRb [18,22,23]. Arginine methylation of E2F-1 by PRMT5 regulates its biochemical and functional properties, including growth control. PRMT5 depletion increases E2F-1 protein expression and is associated with increased apoptosis [24]. In line with this study, we showed that the inhibition of PRMT5 activity in A549 cells by treatment with 10 µM AMI-1 reduced cell viability, induced growth arrest, and apoptosis.

The methylation of histone arginine residues by PRMT5 is a well-known mechanism of transcriptional repression. Among the type II PRMT family members, PRMT5 is the sole member capable of targeting histones [24] and is more commonly associated with transcriptional repression [24,25]. PRMT5 preferentially methylates the N-terminal tails of histones with primary methylation sites being H4 arginine 3 and H3 arginine [24]. We demonstrated that combined AMI-1 and cisplatin administration resulted in the marked methylation reduction in histone-4, with no effect on β-catenin. In our study, β-catenin was tested as a potential target of PRMT5. In a previous study on hepatocellular cancer, there was a significant downregulation of β-catenin upon PRMT5 silencing [26].

AMI-1 is one of the first reported small-molecule inhibitors of PRMT activity. It specifically inhibits PRMT-mediated epigenetic modification of arginine methylation without affecting lysine methylation of cellular proteins in vitro [20]. Initially, AMI-1 was used to inhibit type I PRMTs (PRMT1- PRMT4, PRMT6, and PRMT8) in vitro [17]. However, further studies showed that the effect of AMI-1 on suppressing the proliferation of solid tumors (e.g., hepatocellular carcinoma, colorectal cancer, and gastric cancer) is mediated by inhibiting type II PRMT5 [26,27]. Most studies that exploited the anti-tumor potential of AMI-1 by targeting PRMT5 used a high dose of AMI-1 (in the mM range) [26,27,28,29]. The highlight of our study is that we demonstrated a potential anti-tumor activity of AMI-1 on A549 cells at a concentration as low as 10 μM. Furthermore, the dose of AMI-1 used in our study was found to be safe to a normal bronchial epithelial cell line. These factors prompted us to focus our attention more on AMI-1. Other inhibitors of PRMT5 have also been identified. YQ36286 was investigated as a first-in-class oral inhibitor of PRMT5, selectivity acting against several histone methyltransferases. The IC_50_ of its enzymatic activity was achieved at low nanomolar concentrations. Selective small molecules PRMT5 inhibitors were also explored recently for anti-lung cancer activity [30]. 

In the current study, we demonstrated that *PRMT5* is highly co-expressed with several functionally related genes including *PRMT1*, which mediates arginine mono- and asymmetric dimethylation of many proteins. It is the main enzyme that mediates the methylation of histone H4, a specific tag for epigenetic transcriptional activation [31]. Cyclin-dependent kinase 4 *(CDK4)* is also co-expressed with *PRMT5*. CDK4 phosphorylates and inhibits members of the retinoblastoma (RB) protein family including RB1 and regulates the cell cycle during G(1)/S transition [32].

The success of cisplatin therapy in clinical practice is limited by the occurrence of innate and acquired drug resistance. The mechanisms underlying cisplatin resistance are multiple. Pre-, post-target, on- and off-target types of resistance have been identified, indicating the lack of a single mechanism-based strategy that may overcome cisplatin resistance [33]. Various proteins, genes, or pathways were found to be involved in resistance against cisplatin [34,35].

c-FLIP (CFLAR) is a regulator of the extrinsic apoptotic pathway that decreases caspase 8 activation. Non-small cell lung cancer (NSCLC) is characterized by the over-expression of FLIP and high cytoplasmic expression is further indicative of poor prognosis [36]. Clinically, c-FLIP has been proposed as a prognostic marker in NSCLC [36] and stage II and III colorectal cancer [37]. Chemotherapeutic agents are known to downregulate the gene and protein expression of CFLAR and silencing it facilitates chemotherapeutic-induced apoptosis. The suppression of c-FLIP expression was also found to render resistant human bladder cancer cells more sensitive to cisplatin treatment [38]. Recent studies have demonstrated PRMT1/5 to fine-tune the degradation of anti-apoptotic protein CFLAR_L_ in human lung cancer cells [39]. In this study, PRMT5 protected NSCLC cells from caspase activation and apoptosis induced by anti-cancer drugs, and knocking down its expression led to CFLAR downregulation and activating chemotherapeutic-induced apoptosis. These studies suggest a plausible explanation for the observed additive effect of PRMT5 inhibitor, AMI-1, on cisplatin-induced cellular apoptosis and cell cycle progression.

One of the limitations of our study is that the effect of targeting PRMT1 by AMI-1 was not investigated. However, previous studies showed that the anti-tumor effect of AMI-1 is rather mediated through PRMT5 [26,27]. Another limitation is to precisely define the subcellular localization of PRMT5, which requires nuclear extraction. Additionally, our in vitro results should be validated in vivo using a xenograft model.

In the current study, PRMT5 inhibitor AMI-1 showed two important properties that make it a potential therapeutic adjuvant to cisplatin to combat chemotherapeutic resistance, namely: increasing G1 cell arrest and induction of apoptosis. Additionally, bioinformatic pathway analyses of differentially expressed genes in PRMT5-silenced A549 cells were in line with our in vitro experiments, revealing an increase in apoptosis and a decrease in DNA damage, cell cycle progression, and retinoblastoma activity pathways. A recent study suggested that acquired resistance of NSCLC cells against cisplatin is the consequence of altered signaling, leading to reduced G1 cell cycle arrest and apoptosis [40]. Thus, AMI-1 has a high potential to overcome cisplatin resistance. Altogether, our results showed a potential antineoplastic activity of PRMT5 inhibitor in lung cancer cells in combination with cisplatin. Further investigations are needed to explore the affected cellular pathways and to identify target genes that underwent epigenetic modification in response to this combination treatment.

## 4. Materials and Methods

### 4.1. Bioinformatics

The association between the mRNA expression level of PRMT5 and clinical outcomes of lung adenocarcinoma and lung squamous cell carcinoma (overall survival) was evaluated using a publicly available tool, KMplot.com. This online tool was build using patients’ data obtained from multiple repositories of caBIG, GEO, and TCGA [41]. In silico bioinformatics were used to identify the major pathway associated with the *PRMT5* gene. The microarray dataset of GSE56757 was obtained from the National Center for Biotechnology Information Gene Expression Omnibus (NCIB GEO, http://www.ncbi.nlm.nih.gov/geo, accessed on 28 December 2020). The differentially expressed genes (DEGs) between PRMT5-silenced and control A549 lung cancer cells were analyzed using the GEO2R online tool (https://www.ncbi.nlm.nih.gov/geo/info/geo2r.html, accessed on 28 December 2020). GEO2R uses LIMMA (Linear Models for MicroArray data) and GEOquery packages from the Bioconductor to perform comparison between the selected groups. To determine the enriched pathways in *PRMT5*-silenced A549 cells, the differentially expressed genes from GSE56757 dataset were explored using WebGestalt online tool (WEB-based GEne SeT AnaLysis Toolkit, https://academic.oup.com/nar/article/47/W1/W199/5494758, accessed on 13 January 2021).

### 4.2. Cells and Treatment Protocol

Three cell lines were used in this study; human lung cancer cell lines A549 (European Collection of Authenticated Cell Cultures, Salisbury, UK, Cat. No. 86012804) and DMS 53 (Sigma. Catalog # 950628 23-1VL) were cultured in RPMI medium supplemented with 10% fetal calf serum, and 1% of antibiotics (penicillin/streptomycin) at 37 °C and 5% CO_2_. HBEpC cells (Cell Applications Inc., San Diego, CA, Cat. No. 502-05A) were maintained in human bronchial epithelial cells medium (Sigma Aldrich) supplemented with 1% antibiotics (penicillin/streptomycin) at 37 °C and 5% CO_2_. Cells were seeded in 100 mm^2^ Petri dishes. When cells reached ~40–50% confluency, they were treated with AMI-1 (Sigma Aldrich, Cat. No. A9239) at various concentrations with or without cisplatin (Sigma Aldrich, Cat. No. C2210000) for 24, 48 and 72 h. The protocol for combination treatment involved treating cells with AMI-1 (10 µM) and cisplatin for 24, 48, and 72 h. Control cultures were treated with equal volumes of DMSO as the vehicle.

### 4.3. MTT Cell Viability Assay

Cell viability percentage was examined using the MTT colorimetric assay (3-(4,5-dimethylthiazol-2-yl)-2,5-diphenyltetrazolium bromide; Sigma-Aldrich) following AMI-1, cisplatin or both treatment(s). Cells at a density of 10^4^ were treated with AMI-1, cisplatin, its combination or vehicle, were cultured in 200 μL of growth medium in 96-well plates and assessed after 24 and 48 h. MTT was added and incubated with cells at 37 °C for 2 h in a humidified incubator at 5% CO_2_. Dimethyl sulfoxide (DMSO) was added to dissolve the MTT formazan product and absorbance was read using a 96-well plate spectrophotometer at 570 nm.

### 4.4. Western Blotting Analysis

Cells were lysed in ice-cold NP-40 lysis buffer (1.0% NP-40, 150 mM of NaCl, 50 mM of Tris-Cl, pH 8.0) containing protease inhibitor cocktail tablets (Cat. No. S8830; Sigma, Germany). The protein concentration of cell lysate was quantified using the standard Bradford method (Cat. No. 500-0006; Bio-Rad, Hercules, CA, USA). An amount of 50 μg of protein lysate aliquots were separated by 12% sodium dodecyl sulfate-polyacrylamide gel electrophoresis (SDS-PAGE) and transferred onto a nitrocellulose membrane (Bio-Rad, USA). A total percentage of 5% skimmed milk powder was used to block the membrane at room temperature for 1 h. The membrane was then washed with Tris-buffered saline with 0.1% Tween and incubated with the following primary antibodies (anti-PRMT-5, anti-β-catenin, anti-H4R3me2s [Symmetrical dimethylation on arginine-3 of histone H4], anti-β-actin (all from Abcam, Cambridge, UK), (cyclin D1, cdk4, cdk6, caspase-3, and PARP, all from Cell Signaling Technology, Danvers, MA, USA) overnight at 4 °C. Secondary antibodies (Cell Signaling Technology, Danvers, MA, USA) were incubated with the membrane at 1:1000 dilution for 1 h at room temperature. Chemiluminescence was detected using the ECL kit (Thermo Scientific Pierce, Rockford, IL, USA). Bio-Rad Image Lab software (ChemiDoc™ Touch Gel and Western Blot Imaging System; Bio-Rad, Hercules, CA, USA) was used to detect and quantify protein bands. Protein levels were normalized to β-actin and ratios were calculated based on the values of control (untreated) samples.

### 4.5. Immunofluorescence Staining

Cells were seeded at a density of 5 × 10^5^ cells/mL; at around 60% confluency, cells were treated as indicated above. Cells were washed twice with PBS, fixed with (PERM/FIX buffer from BD) and cells were stained with anti-PRMT-5 (Abcam, Cambridge, UK), overnight at 4 °C. Cells were then washed with 1× PBS and reacted with the Alexafluor^®^488-labeled secondary antibody (Abcam, Cambridge, UK) for 1 h at 37 °C; excess reagent was rinsed with 1× PBS. Genomic DNA was stained with 4′,6′-diamidino-2-phenylindole (DAPI) (Invitrogen, Carlsbad, CA, United States) according to manufacturer’s instructions. Slides were visualized by confocal microscopy using a Nikon Confocal Microscope (Nikon, Tokyo, Japan). PRMT5 subcellular localization (nuclear and cytoplasmic) were quantified by measuring fluorescence intensity profiles using Image J software, National Institutes of Health (NIH), USA (http://rsb.info.nih.gov/ij/index.html, accessed on 2 April 2021). A total of 30 cells per experiment were analyzed under 40× objective lens and all images were acquired using identical parameters. 

### 4.6. Cell Cycle Progression Analysis

Cell cycle progression was analyzed by staining the cells with the Propidium Iodide Flow Cytometry Kit (Abcam, Cambridge, UK). According to the manufacturer protocol, 1 × 10^6^ cells were seeded and treated as indicated above. Harvested cells were washed twice with PBS, fixed in 70% ethanol, and stored at −20 for 48 h. After pelleting the cells, they were washed twice with PBS then incubated with staining buffer containing propidium iodide (PI) and RNase in the dark at room temperature for 15 min. The cell cycle profile of different phases was examined by flow cytometry (Accuri C6; Becton, Dickinson and Company, Franklin Lakes, NJ, USA). Cell cycle analysis of cell percentages occupying sub-G1, G1, S, and G2/M phases of the cell cycle were determined using the cell cycle platform of the FlowJo software with the Watson pragmatic model (Tree Star).

### 4.7. Annexin-V Staining for Apoptosis Detection

Apoptosis induction was assessed using the Annexin V-FITC Apoptosis Detection Kit protocol (Abcam, Cambridge, MA, USA). Briefly, 1 × 10^6^ cells were seeded and treated as indicated above. Harvested cells were washed twice with PBS and incubated with staining buffer that contained annexin-V/PI for 20 min in the dark at room temperature. Cells were then analyzed for apoptosis by flow cytometry (Accuri C6; Becton Dickinson and Company) at 488/530 nm excitation. Cells negative for both dyes were considered living, PI-positive staining only as necrotic, positive for annexin V staining only as early apoptotic, annexin V/PI-positive as late apoptotic. Flow cytometric acquired data were analyzed using the FlowJo software with the Watson pragmatic model (Tree Star, Ashland, OR, USA).

### 4.8. Statistical Analysis

Each experiment was performed at least three times. The results were expressed as mean ± standard deviation (SD). Two-way analysis of variance (ANOVA) with Tukey’s multiple comparisons test was used for multiple comparisons of values. *p*-values < 0.05 was considered statistically significant. For detecting the significantly different groups, we used the Bonferroni test. Data fitting and graphs were presented using the GraphPad Prism 8 software (San Diego, CA, USA).

## 5. Conclusions

The findings of the current study indicate that PRMT5 inhibition using AMI-1 has an additive effect on the antineoplastic activity of cisplatin in lung cancer cells. Overall, AMI-1 may overcome the cisplatin resistance by arresting cancer cells and inducing apoptosis. AMI-1 did not show toxicity in normal bronchial epithelial cells. Therefore, AMI-1 is a promising therapeutic candidate in improving cisplatin resistance in lung cancer.

## Data Availability

Data sharing is not applicable to this article.

## Abbreviations

PRMT5	Protein arginine methyltransferase 5
HBEpC	Human bronchial epithelial cells
AMI-1	Arginine methyltransferase inhibitor 1
MTT	3-(4:5-dimethylthiazol-2-yl)-2,5-diphenyltetrazolium bromide
SDS	sodium dodecyl sulfate-polyacrylamide
H4R3me2s	Symmetrical dimethylation on arginine-3 of histone H4
DMSO	Dimethyl sulfoxide
PI	propidium iodide
OS	Overall survival
HR	Hazard ratio
CI	Confidence interval
